# Comment on van Netten, et al: Definitions and criteria for diabetic foot disease

**DOI:** 10.1002/edm2.142

**Published:** 2020-05-10

**Authors:** Gustav Jarl, David F. Rusaw, Anton Johannesson

**Affiliations:** ^1^ Department of Prosthetics and Orthotics Faculty of Medicine and Health Örebro University Örebro Sweden; ^2^ University Health Care Research Center Faculty of Medicine and Health Örebro University Örebro Sweden; ^3^ School of Health and Welfare Jönköping University Jönköping Sweden; ^4^ International Organization for Standardization Sibbhult Sweden; ^5^ Össur Clinics Scandinavia Stockholm Sweden

**Keywords:** amputation, classification, diabetic foot

## Abstract

**Objective:**

The International Working Group on the Diabetic Foot (IWGDF) recently published updated definitions for the diabetic foot field. However, the suggested definitions of lower limb amputations differ from the definitions of the International Organization of Standardization (ISO), which may create problems when implementing the definitions. This paper compares and discusses the amputation definitions of IWGDF and ISO.

**Results:**

Despite many similarities, the IWGDF and ISO systems have some important differences. First, the IWGDF uses the term “minor amputation” which is value‐laden, arbitrary and has been defined in several different ways in the literature. Second, the IWGDF system lacks descriptions of amputations distal or through the ankle, which may increase the risk for misclassification. Third, hip disarticulations and transpelvic amputations are not included in the IWGDF system.

**Conclusion:**

It is suggested that future updates of the IWGDF definitions should be aligned with those of ISO, to meet the goal of global consensus on terminology related to lower limb amputation.

## Graphical abstract

The International Working Group on the Diabetic Foot (IWGDF) recently published an update of definitions for the diabetic foot field. However, the suggested definitions of lower limb amputations differ from the definitions of the International Organization of Standardization (ISO), which may create problems when implementing the definitions. This paper compares and discusses the definitions of IWGDF and ISO and suggests that future updates of the IWGDF definitions should be aligned with those of ISO, to meet the goal of global consensus on terminology related to lower limb amputation.

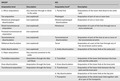

## 1 INTRODUCTION

The International Working Group on the Diabetic Foot (IWGDF) has created and published definitions related to diabetic foot disease since 1999. The latest update^1^ was published this year in Diabetes/Metabolism Research and Reviews and includes definitions related to diabetic foot disease, the foot, foot ulceration, peripheral arterial disease, infection, amputation and other miscellaneous definitions. These definitions are important contributions to the diabetic foot field to facilitate communication and comparisons of results from audits and research studies. However, as the IWGDF's categorization of amputations differs from the International Organization for Standardization (ISO),^2^ it is not clear how to categorize amputations. This has the potential to create problems when utilizing the classifications to facilitate comparison and communication.

## 2 COMPARISON OF IWGDF AND ISO DEFINITIONS AND CATEGORIZATIONS OF LOWER LIMB AMPUTATIONS

The IWGDF and ISO systems have many similarities, for example the general definitions of amputation are very similar, with IWGDF defining it as ‘resection of a segment of a limb through a bone or through a joint’, and ISO defining it as ‘surgical removal of the whole or part of a limb’. Additionally, most lower limb amputation levels can be found in both classification systems (Table 1). Despite these similarities, there are some important differences. First, the IWGDF categorizes amputations into two groups: major amputations and minor amputations. This traditional dichotomization has received critique over the years, among other things, for being value‐laden: a minor amputation may not be experienced as minor by the patient. Also, the distinction between major and minor amputations is somewhat arbitrary as certain levels of partial foot amputations (minor amputations) can have similar effects as a transtibial amputation (major amputation) on gait characteristics, energy expenditure and quality of life.^3^ Furthermore, minor amputations are defined differently in the literature, for example as amputations distal to the tarsometatarsal joint,^4^ distal or through the tarsometatarsal joint,^5^ distal to the ankle,^6^ or distal or through the ankle.^1^ This previous point is particularly problematic for comparison and communication, as the result is two groups (major/minor) which cannot be guaranteed as mutually exclusive. Second, the IWGDF system lacks descriptions of amputations distal or through the ankle (Table 1), which may increase the risk for misclassification. For example, surgical removal of a whole digit may be misclassified as a toe amputation, when the correct classification is metatarsal‐phalangeal disarticulation. Third, hip disarticulations and transpelvic amputations are not included in the IWGDF system. This may be due to the fact that amputations at these levels are uncommon and, in most cases, not related to diabetes.^7^, ^8^, ^9^, ^10^ Regardless, it could be seen as counterproductive to use one system to categorize amputations related to diabetes, and another system to categorize other amputations. One may object that the ISO system also lacks amputation levels, as the IWGDF differentiates between proximal and distal transmetatarsal amputations, which the ISO does not. However, this is a deliberate choice of ISO, stating that for all partial foot amputations the complete description requires ‘the identification of the amputated bones and their levels of amputation’, as this level of amputation can include all, some or a variety of skeletal structures.

**Table 1:** Comparison of definitions for amputation levels of the lower limb

IWGDF		ISO	
Amputation level	Description	Amputation level^a^	Description
1. Minor amputations	Any resection through or distal to the ankle	1. Partial foot	Amputations of the lower limb distal to the ankle joint
Toe amputation	(not explained)	Phalangeal	Amputation of part of one or more toes
Metatarsal‐phalangeal disarticulation	(not explained)	Metatarsophalangeal disarticulation	Amputation of one or more toes
Distal transmetatarsal amputation	(not explained)	Metatarsal	Amputation of a part of the foot through one or more metatarsals
Proximal transmetatarsal amputation	(not explained)		
Tarsometatarsal disarticulation	(not explained)	Tarsometatarsal disarticulation	Amputation of part of the foot at one or more of the tarsometatarsal joints
Midtarsal disarticulation	(not explained)	Tarsal	Amputation of a part of the foot through any of the tarsal bones and/or joints.
Ankle disarticulation	(not explained)	2. Ankle disarticulation	Amputation of the lower limb at the ankle joint
2. Major amputations	Any resection proximal to the ankle		
Transtibial amputation	Amputation through the tibia and fibula	3. Transtibial	Amputation of the lower limb between the knee joint and the ankle joint
Knee disarticulation	Amputation through the knee	4. Knee disarticulation	Amputation of the lower limb at the knee joint
Transfemoral amputation	Amputation through the femur	5. Transfemoral	Amputation of the lower limb between the hip joint and the knee joint
		6. Hip disarticulation	Amputation of the lower limb at the hip joint
		7. Transpelvic	Amputation of the whole lower limb together with all or part of the hemipelvis

Note

IWGDF, International Working Group on the Diabetic Foot; ISO, International Organization for Standardization.

^a^ During 2020, the revised version of ISO 8548‐2 (Method of describing lower limb amputation stumps) and ISO 8549‐2 (Prosthetics and orthotics—Vocabulary—Part 2: Terms relating to external limb prostheses and wearers of these prostheses) will be published where the hyphen has been removed when describing amputation levels. Other ISO standards, within this working group, are up for revision every 5th year. Suggestion for changes can be send to the Secretary of ISO working group 168 during that period.

## 3 DISCUSSION

The IWGDF definitions are a valuable source for clinicians and researchers working with diabetic foot disease, bringing clarity to the diverse terminology of this multidisciplinary field. However, as different international initiatives to standardize may overlap, it is important to compare suggested definitions with those already existing in the field and, when appropriate, harmonize definitions across standardization systems. For example, for diagnosis of foot infection, the IWGDF is aligned with the Infectious Diseases Society of America (IDSA).^11^ We agree and would welcome a similar approach in future updates of the IWGDF definitions, suggesting that the definitions of amputations be aligned with those from ISO. By doing so, we can ensure that the goals of these classification systems (to facilitate communication and comparison) are in alignment and that both systems are of maximum benefit to those working in the multiple fields that make use of these systems.

## CONFLICT OF INTEREST

GJ is a consultant for Novo Nordisk and AJ is an employee of Össur Clinics Scandinavia. DFR has no conflicts of interest to disclose.

## AUTHORS CONTRIBUTIONS

GJ wrote the first draft. All authors have contributed to writing, discussions of the content, and have read and approved the final manuscript.

## ETHICS STATEMENT

Not applicable.

## Data Availability

Not applicable.
